# Multidisciplinary Management of Soft Tissue Sarcoma

**DOI:** 10.1155/2013/852462

**Published:** 2013-07-28

**Authors:** Lukas M. Nystrom, Nickolas B. Reimer, John D. Reith, Long Dang, Robert A. Zlotecki, Mark T. Scarborough, C. Parker Gibbs

**Affiliations:** ^1^Orthopaedic & Sports Medicine Institute, University of Florida, 3450 Hull Road, Gainesville, FL 32605, USA; ^2^Department of Pathology, Immunology and Laboratory Medicine, University of Florida, 1600 SW Archer Road, Gainesville, FL 32608, USA; ^3^Department of Medicine, Division of Hematology & Oncology, University of Florida, 1600 SW Archer Road, Gainesville, FL 32608, USA; ^4^Department of Radiation Oncology, University of Florida, 1600 SW Archer Road, Gainesville, FL 32608, USA

## Abstract

Soft tissue sarcoma is a rare malignancy, with approximately 11,000 cases per year encountered in the United States. It is primarily encountered in adults but can affect patients of any age. There are many histologic subtypes and the malignancy can be low or high grade. Appropriate staging work up includes a physical exam, advanced imaging, and a carefully planned biopsy. This information is then used to guide the discussion of definitive treatment of the tumor which typically involves surgical resection with a negative margin in addition to neoadjuvant or adjuvant external beam radiation. Advances in imaging and radiation therapy have made limb salvage surgery the standard of care, with local control rates greater than 90% in most modern series. Currently, the role of chemotherapy is not well defined and this treatment is typically reserved for patients with metastatic or recurrent disease and for certain histologic subtypes. The goal of this paper is to review the current state of the art in multidisciplinary management of soft tissue sarcoma.

## 1. Epidemiology

Soft tissue sarcomas are a relatively rare and heterogeneous group of malignancies that are characterized by mesodermal differentiation [1]. They occur in various anatomic locations in addition to the extremities, including the chest wall, retroperitoneum, and head/neck, among others. Extremity soft tissue sarcomas exhibit numerous histologic subtypes and may be low or high grade and subcutaneous or deep in location. The vast majority metastasize hematogenously, though select subtypes can also spread through the lymphatic system. In the United States in 2005 an estimated 3490 deaths were related to soft tissue sarcoma [2], less than 1% of all cancer-related deaths.

It was estimated that in 2012 there would be 11,280 new cases of soft tissue sarcoma in the United States, making the diagnosis approximately three to four times more common than primary bone sarcoma [3]. The international incidence is estimated to be approximately 1.8 to 5 per 100,000 people per year [4]. Of the over 50 different histologic types of soft tissue sarcoma, the most common is undifferentiated pleomorphic sarcoma (UPS) [5]. Soft tissue sarcomas can occur at any age; however, most arise in patients over the age of 55 [5] and are usually UPS or liposarcoma. Those occurring in patients younger than 20 are more likely to be rhabdomyosarcoma or synovial sarcoma [6, 7]. Epithelioid sarcoma also is more common in younger adults. Males are affected slightly more frequently than females.

Optimal management of soft tissue sarcoma relies upon an appropriately performed biopsy, accurate diagnosis and staging, an effective surgical plan and execution, rational utilization of adjuvant therapies, and close surveillance following resection. This is best carried out at a tertiary care center with an experienced multidisciplinary team specializing in the care of sarcoma patients. The purpose of this paper is to review the principles and issues surrounding the diagnosis and treatment of soft tissue sarcoma.

## 2. Diagnosis and Staging

### 2.1. History and Physical Examination

The typical presenting complaint of a patient diagnosed with soft tissue sarcoma is that of an enlarging mass. Characteristics such as size greater than 5 cm, location deep to fascia, and rapid tumor growth are worrisome and should raise suspicion of a sarcoma [8, 9].

Appropriate workup of a suspected sarcoma should begin with a careful history and physical examination. Important elements of the history are duration of mass, rate of growth, pain, weakness or numbness, history of trauma, exposure to radiation or other carcinogenic toxins, personal or family history of cancer, and smoking history. The examination should note the characteristics of the size and consistency of the mass, presence of pain with palpation, its anatomic compartment and location relative to the fascia and neurovascular structures, regional lymph node examination, and neurovascular examination of the affected extremity. Additionally, the mass should be evaluated for the presence of a bruit or thrill and the presence of a tinel's sign. The biopsy of a lesion with an unappreciated vascular or neural component can result in copious bleeding or neurologic embarrassment. Specific attention should be paid to overlying skin lesions that might suggest an underlying disease state, such as neurofibromatosis heralded by café au lait spots. Along this line, one should consider malignant peripheral nerve sheath tumor as the primary working diagnosis in a patient with an enlarging painful mass in the setting of neurofibromatosis. 

### 2.2. Imaging

Radiographs of the affected extremity should be obtained and scrutinized for the presence and size of a soft-tissue shadow, bony destruction, and intratumoral calcifications. Furthermore, radiographs can help identify a primary bone tumor associated with a large soft tissue mass such as is often seen in Ewing's sarcoma. The amount and type of bony destruction can provide evidence of the biologic activity of the tumor. Slow growing masses may exert a pressure effect with cortical remodeling marked by a well-defined reactive rim. More rapidly growing masses may produce a more irregular cortical destruction. While phleboliths are a finding in benign hemangiomas, amorphous calcification is a characteristic of synovial sarcoma [5] and some liposarcomas. 

Magnetic resonance imaging (MRI) is the modality of choice for evaluation of a potential soft tissue sarcoma, both for diagnostic characterization and staging purposes to plan effective management. If MRI is not feasible due to an incompatible medical device or high-risk metallic foreign body, a computerized tomography (CT) scan with and without intravenous contrast is recommended with 3D reconstruction so as to assess local extent of disease in longitudinal and axial planes. The MRI exam should be performed with and without intravenous gadolinium enhancement [8]. A soft tissue sarcoma will typically exhibit heterogeneous high-signal intensity on T2 weighted images. There may also be substantial peritumoral edema. T1 weighted images best demonstrate normal anatomy and its relation to the tumor and typically is relied upon for preoperative planning. As the sequence is not fluid sensitive, it will help differentiate tumor from the edema seen on T2 sequences. Often the delineation between tumor tissue and uninvolved tissue is further defined by T1 postgadolinium images. Occasionally, the tumor extent can be overestimated on T2 sequences as the fluid sensitive nature of the sequence cannot easily define edema as opposed to actual tumor extent. 

Dynamic Contrast Enhanced MRI (DCE-MRI) is a method of physiologic imaging that uses bolus administration of water soluble and paramagnetic contrast, rapidly obtained sequences, and sophisticated software to measure perfusion, and diffusion characteristics of the imaged tissue [12]. DCE-MRI relies on the contrast agent first passing through the capillaries of the tumor and then rapidly diffusing into the interstitial compartment. This essentially exploits the increased vascular density, high perfusion and increased permeability associated with soft tissue sarcomas. Viable tumor enhances rapidly and areas of necrosis, degeneration, hemorrhage, and fibrosis enhance much more slowly. The major advantage of this form of imaging is that it has the ability to potentially reflect tumor biology. It can potentially define the most aggressive part of a tumor for biopsy, determine response to chemotherapy, and differentiate recurrence from inflammatory tissue [12].

In addition to cross-sectional imaging of the mass, additional staging studies of a suspected or diagnosed soft tissue sarcoma should include a CT scan of the chest to evaluate for pulmonary metastases. We recommend obtaining this on initial presentation and following any neoadjuvant therapy, as the finding of metastatic disease may alter the goals of the surgical treatment plan. Bone scans are seldom used for staging purposes as the incidence of bone metastasis is extremely low.

**Figure 1:**
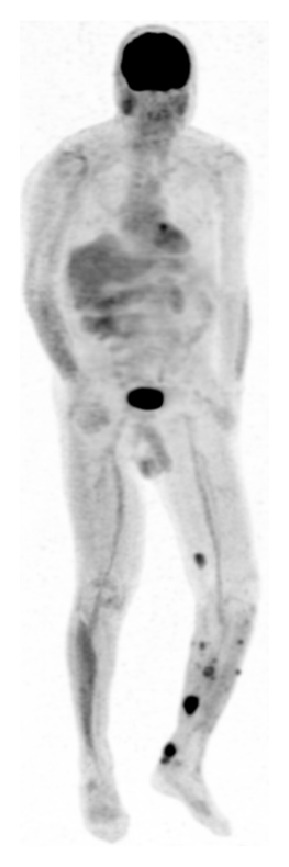
PET scan obtained to evaluate and stage the extent of histologically confirmed local recurrence of left lower leg soft tissue sarcoma.

The role of positron emission tomography (PET) scanning is evolving and has shown promise in the area of soft tissue sarcoma [13, 14]. PET scan technology assesses the in vivo metabolic activity via positron-emitting radionuclides. Fluorodeoxyglucose-18 (FDG-18) is the most commonly used radionuclide; however, there are several under investigation. FDG-18 is a glucose analog that is actively transported into cells where it is intended as a substrate for metabolism. Once in the cell, it is phosphorylated to glucose-6-phosphate analog and becomes trapped as this analog cannot be utilized for glycolysis. The tagged radionuclide then undergoes positron decay and the positrons collide with electrons to create photons that travel in opposite directions where they are detected by the PET scanners. Software programs then incorporate information about tracer quantity and patient body weight to calculate maximum and standardized uptake value (SUV) [5, 13]. Furthermore, PET scan images are often fused with cross-sectional CT imaging (PET/CT) to provide functional and anatomical imaging. There have been several applications of FDG-PET to soft tissue sarcoma including grading of tumors, initial staging [15], assessing response to neoadjuvant therapy [16], determining prognosis [16, 17], and investigating potential local recurrence [18] (Figure 1). In a recent publication evaluating the diagnostic and prognostic value of the PET/CT in sarcoma, it was found that the test is highly sensitive and specific in the detection of high-grade bone and soft tissue sarcoma but had a low positive predictive value for evaluation of nodal metastases [19]. Furthermore, it was found that the SUVmax of the primary tumor was a strong predictor of survival [19]. 

Currently, the United States Food and Drug Administration has approved the use of FDG-PET for diagnostic purposes in epilepsy, left ventricular dysfunction, and staging of cancer. Medicare reimbursement covers the test for diagnosis, staging, and restaging of lymphoma and melanoma, colorectal, nonsmall cell lung, breast, head/neck, esophageal and cervical cancers, and for differentiation of solitary pulmonary nodules. Although it has demonstrated potential benefit, the use of FDG-PET in soft tissue sarcomas is still considered investigational. Further areas of investigation include fusing PET images with MRI (PET/MRI) to provide enhanced functional/anatomic visualization for staging and evaluation of recurrence in soft tissue sarcomas.

### 2.3. Biopsy

**Figure 2:**
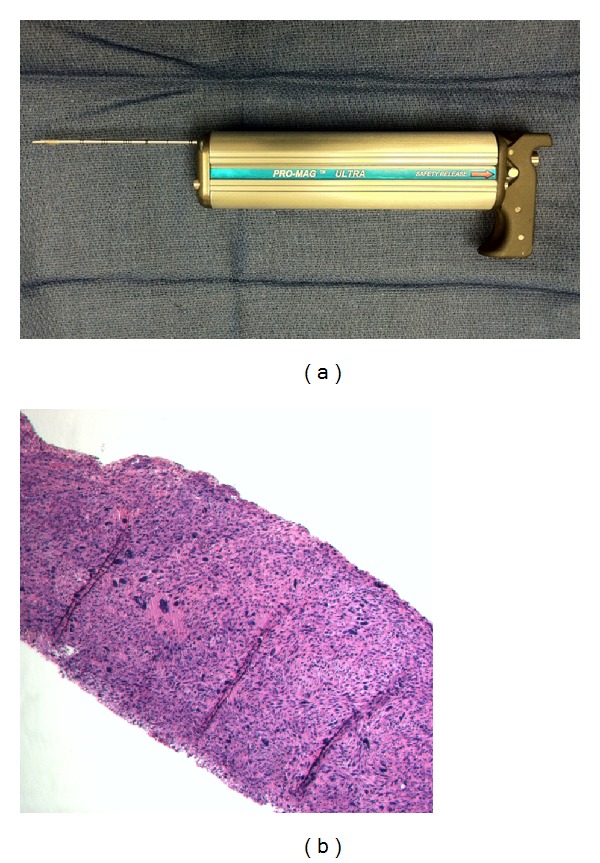
(a) Tru-Cut core needle biopsy device (Dyna Medical, Ontario, Canada). The device functions by discharging a spring-loaded hollow bored needle a distance of 2 cm into the targeted tissue to obtain the core specimen (b).

Although not technically challenging, the planning and execution of the biopsy of a suspected soft tissue sarcoma should be performed with care and consideration of the definitive resection procedure. For most soft tissue masses, a core needle biopsy (Figure 2(a)) is preferred as it is minimally invasive and can be performed in clinic. Unlike fine needle aspirates, the core biopsy preserves tissue architecture for the pathologic examination (Figure 2(b)). Core needle biopsy has been shown to have a diagnostic accuracy of 84–90% [20, 21]. Furthermore, the ability to perform this biopsy in the clinic represents a substantial cost savings over open incisional biopsy to the patient and health care system [21]. If desired, ultrasound can potentially assist in avoiding vital anatomic structures or nonviable areas of tumor; however, we find that this is generally not necessary.

If an incisional biopsy is required for patient- or tumor related factors, it should be performed in line with the skin incision of the planned resection of the malignant lesion. Meticulous hemostasis should be obtained so as to prevent contamination by hematoma. If a drain must be used, it should exit in line with the skin incision as close as possible to avoid potential regional contamination. The hazards of an inappropriately executed biopsy are well documented in the literature [22, 23]. A 1996 study reported that biopsies performed prior to referral to the treating center resulted in a two to twelve times greater rate in diagnostic errors, complications, and changes in the course and outcome than when the patient was referred to a sarcoma treatment center prior to biopsy [23]. Optimally, the biopsy is performed by the surgeon who will perform the definitive resection.

Certain histologic subtypes of soft tissue sarcoma are known to have a higher predilection for nodal metastasis, especially synovial sarcoma, epithelioid sarcoma, rhabdomyosarcoma and clear cell sarcoma [24–26]. The rate of metastasis to lymph nodes is estimated at approximately 6% [24]. If one of these diagnoses is identified on biopsy a careful clinical exam and review of imaging for enlarged nodes should be performed. If an enlarged node is identified a sentinel lymph node biopsy may be considered at the time of the resection, although it is unclear whether this improves long-term survival [26, 27]. A 2013 study by Sawamura et al. comparing patients with nodal metastases treated with and without lymphadenectomy noted improved survival at 1.5 years in those managed with lymphadenectomy but subsequently no difference at 5 years [27].

### 2.4. Histologic Analysis and Tissue Handling

All biopsy specimens, regardless of type, should be submitted fresh to the pathology laboratory for frozen section evaluation. This practice will assure that viable diagnostic tissue is present in the biopsy specimen and that the tissue can be appropriately triaged for ancillary diagnostic tests. Ideally, the surgeon and the pathologist should discuss the differential diagnosis and review the imaging prior to the biopsy to maximize diagnostic success. The frozen section interpretation will generally fall into one of three categories: (1) the tissue is diagnostic of a specific lesion that will likely not require further testing (e.g., pleomorphic liposarcoma); (2) diagnostic tissue is present, but further ancillary testing is necessary for a specific diagnosis (e.g., a small round blue cell neoplasm); or (3) the tissue is not diagnostic, in which case the managing clinician will need to perform additional biopsies [28, 29].

The proper triage of tissue at the time of frozen section evaluation is an important step in the diagnostic workup of any soft tissue sarcoma. In addition to routine histologic and immunohistochemical studies, flow cytometry (used to evaluate possible lymphomas), cytogenetics, and molecular/molecular cytogenetic tests are routinely performed for diagnosis; particularly for sarcomas with a well-established chromosomal rearrangement. In particular, many molecular and molecular cytogenetic tests are routinely utilized for the diagnosis of soft tissue sarcomas, and while the majority of these tests can now be performed on paraffin-embedded tissue, it is good practice to assure that sufficient volume of viable tissue is available for such studies. 

When possible, soft tissue sarcomas should be classified using the nomenclature of the World Health Organization [30]. However, occasionally soft tissue sarcomas cannot be easily classified into a specific histologic subtype. From a clinical standpoint this places increased importance in assigning an accurate histologic grade. The most commonly used sarcoma grading system is a three-tiered system proposed by the French Federation of Cancer Centers [31], which considers the parameters of tumor differentiation, mitotic activity, and necrosis in formulating an overall grade of low, intermediate, or high. A second three-tiered system has been proposed by the National Cancer Institute [32] which involves more parameters than the French system. For practical purposes, including integration of histologic grade into the surgical staging system described by Enneking et al., a two-tiered grading system may be more practical and reproducible. Whichever grading system is employed, accurate grading is an important exercise for both treatment decisions and as a prognostic variable. 

Following resection of a soft tissue sarcoma, the role of the pathologist is essentially four-fold: confirm the biopsy diagnosis, provide grading information for staging, evaluate margins and assess the response of the tumor to neoadjuvant radiation or chemotherapy. Margins are classified as intralesional, marginal, wide, or radical depending on the quality of the tissue at the resection margin. This is discussed in more detail in the surgical treatment section. Margins can be assessed in a variety of manners; however, a combination of gross inspection and frozen section analysis is often utilized intraoperatively to assure negative margins [33], and then confirmed with permanent sections following formal dissection of the specimen. Assessment of tumor response to neoadjuvant therapy is of debatable significance with soft tissue sarcomas; however, the processing of soft tissue sarcomas in a manner similar to that utilized for osteosarcomas will eliminate sampling bias and assure accurate assessment of the response.

### 2.5. Staging Systems

Assigning a stage to a patient with sarcoma is a process that involves compiling all of the data from the above mentioned clinical, radiographic and histologic examinations. The process is informative to the prognosis of the patient but also allows for effective study of treatment and outcomes of patients with similar tumor characteristics. Although none of the staging systems have been validated in large groups of patients, the factors that are felt to carry the most importance are the size of the tumor, anatomic location, the presence of nodal and/or metastatic lesions and the tumor grade. These are represented in the system from the American Joint Commission on Cancer (AJCC) [10]. Although, compartmental extent has not been shown definitively to affect prognosis, it is widely accepted as an important surgical consideration and is represented in the system described by Enneking et al. and adopted by the Musculoskeletal Tumor Society (MSTS) [11]. These staging systems are summarized in Table 1.

## 3. Treatment

### 3.1. Radiation Therapy

**Figure 3:**
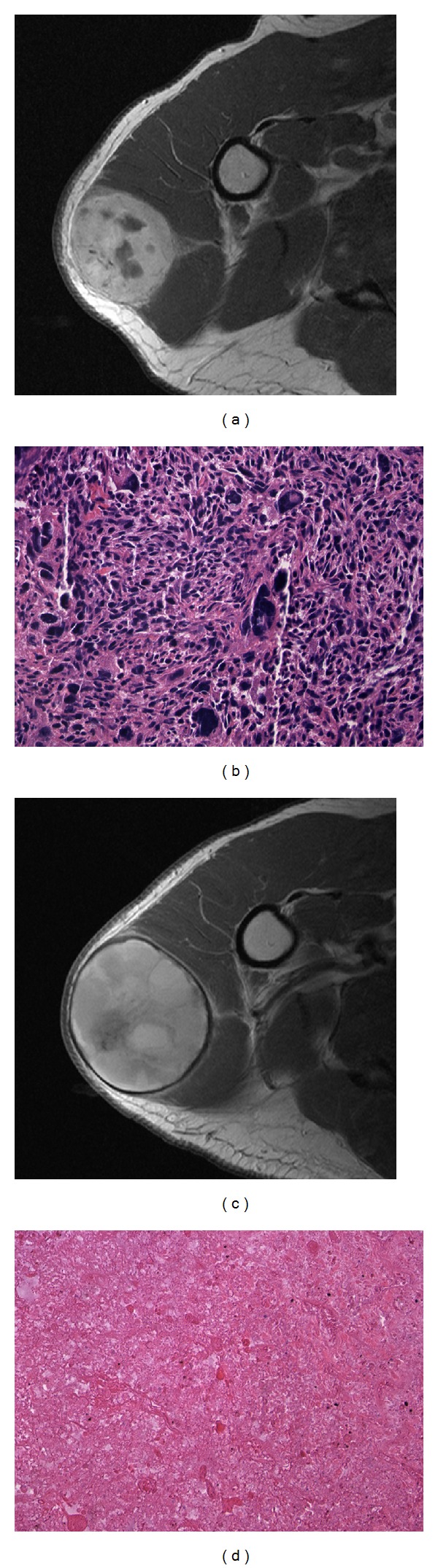
(a) Pre-operative axial T1 post-contrast MRI demonstrating a large, deep, soft tissue sarcoma contained within the deltoid musculature, and (b) the core needle biopsy specimen (Frozen Section, H&E, 20x) demonstrating high-grade undifferentiated pleomorphic sarcoma. (c) The same tumor on a restaging axial T1 MRI after pre-operative radiation demonstrating a thick fibrous rind. Also evident is that the tumor has grown slightly with radiation. (d) Histopathologic slide (H&E, 20x) from the resection specimen demonstrating substantial treatment effect from the radiation.

The benefit of adjuvant radiation therapy has been clearly demonstrated in the treatment of soft tissue sarcomas. In general, a standard dose of preoperative radiation involves 50 Gy delivered over a 5 week period. Surgery then follows after a 3-4 week “rest” period to allow the overlying soft tissues to heal. Postoperative radiation doses are higher, approximately 65 Gy delivered over 6-7 weeks, and are usually delivered after the wound has been determined to heal (usually at 3–6 weeks postoperatively). When combined with surgery with negative margins local control rates has been reported to be 90% or greater. The effect of radiation is believed to be exerted by sterilization of the tumor capsule by killing the microscopic extensions of the tumor. This both decreases the intrinsic risks of local recurrence, and also permits the sparing of critical normal tissue structures with focal marginal resection planes. From a surgical standpoint this often manifests in the formation of a fibrous rind or capsule surrounding the tumor, which is often evident on MRI (Figure 3). Radiation can be delivered either pre- or post-operatively. Both alternatives have benefits and drawbacks.

#### 3.1.1. Preoperative versus Postoperative Radiation

The benefits of preoperative radiation include equivalent treatment effect with delivery of a smaller total dose and exposure of a smaller volume of normal tissue to irradiation. This is particularly important when considering that these two factors involving radiation have been shown to have an effect on joint stiffness and radiation fibrosis, impacting functional outcome [34]. Furthermore, increased radiation dose has been correlated with risk of secondary malignancies. The main disadvantage of pre-operative radiation is a higher risk of acute wound healing complications. In the 2002 study by O'Sullivan et al. they demonstrated that 35% of patients receiving preoperative radiation therapy, versus 17% receiving postoperative radiation therapy developed wound complications [34]. In that study there was no significant difference in rates of local recurrence, metastases or progression free survival between the two groups. There was, however, a statistically significant difference in overall survival, favoring preoperative radiation, at the time of last follow-up (median 3.3 years) [34]. This small survival benefit noted in this group, however, must be interpreted with caution as the deaths in the post-operative group did not seem to be related to progression of the sarcoma alone [34].

#### 3.1.2. Effect of Radiation on Margins

An investigation into the margin status with regards to limb-sparing surgical resections was published in 2012 by Dagan et al. [35]. In that study a retrospective review of 317 patients with non-metastatic extremity soft tissue sarcoma evaluated the margins, according to the Enneking classification [11], at the time of a limb sparing surgical resection. Margin status was then correlated with local recurrence, amputation free survival, cause specific survival and overall survival. The authors reported that the five year local control rates were equivalent at 95% for both marginal and wide/radical margin resections. The five year amputation free survival was 97% and 92%, respectively. The authors concluded that when preoperative radiation is incorporated into the patient's care excellent rates of local control are obtained regardless of whether they have marginal, wide or radical resections [35]. Studies of postoperative radiation have found a similar effect on local control [36].

### 3.2. The Role of Chemotherapy

The role of chemotherapy in the treatment of STS is controversial. In general, the regimens are highly toxic and have failed to show long term survival benefits. As individual studies had not shown conclusive benefit, a meta-analysis of 1568 patients was undertaken by the Sarcoma Meta-Analysis Collaboration and published in a classic article from 1997 [37]. In that study of doxorubicin-based chemotherapy regimens combined with surgery for local control the investigators demonstrated statistically significant absolute benefits with regard to local recurrence at 6% and distant recurrence at 10%, at 10 years. However, when overall survival was analyzed the authors were unable to show a statistically significant benefit to improved survival at 10 years [37]. The article from the Sarcoma Meta-Analysis Collaboration has been criticized for including patients with primary and recurrent disease, extremity and non-extremity tumors, lack of complete data on tumor grade and size and significant tumor heterogeneity [38].

A prospective randomized controlled trial published in 2001 by the Italian randomized cooperative trial compared 104 patients randomized to no chemotherapy or chemotherapy with ifosfamide and epidoxorubicin [39]. Inclusion criteria for their trial were high-grade, deep, extremity tumors greater than 5 cm with no prior chemotherapy. Patients with metastatic disease were excluded. All patients were also treated with pre- or post-operative radiation and resection. These investigators found that at two years chemotherapy conferred a 13% absolute survival benefit when compared to the control arm (85% versus 72%), and that this survival benefit increased to 19% at four years (69% versus 50%) [39]. The study was stopped after an initial analysis yielding these results. However, the same cohort was revisited in a 2003 study to update the results at a median follow-up of 89 months. The results of this follow-up study were that the advantages in disease free survival and overall survival were no longer statistically different at this time point [40]. 

A combined retrospective study from the Memorial Sloan-Kettering Cancer Center and University of Texas M.D. Anderson Cancer Center evaluated 674 consecutive adult patients with stage III extremity soft-tissue sarcoma treated with and without chemotherapy [38]. This study demonstrated measure statistically significant benefits favoring the group treated with chemotherapy in terms of local control, metastases, overall disease free survival and disease specific survival at one year. However, these benefits were not maintained at 5 year follow-up [38]. Based on the findings of this study these investigators concluded that patients be informed the initial benefits of chemotherapy may not be maintained over time and that literature reporting short term outcomes of soft tissue sarcoma treated with chemotherapy be interpreted with caution.

Thus far the majority of studies regarding chemotherapy regimens have included all of the various histologic subtypes of soft-tissue sarcoma in one analysis. This is largely due to the relative rarity of the disease as a whole. It has been argued that the most effective way to advance adjuvant treatment is by studying histologic subtypes individually and targeting specific gene products [41]. There are two histologic subtypes of soft tissue sarcoma which have demonstrated a more favorable response to chemotherapy: synovial sarcoma and pediatric rhabdomyosarcoma. These tumors also have known chromosomal translocations, which offer potential for specific targeted therapeutic regimens [41]. A retrospective study comparing the behavior of synovial sarcoma has shown that it responds more favorably to adjuvant chemotherapy with a five year metastasis free survival rate of 60% compared to 48% in those that did not receive chemotherapy [42]. The largest benefit was seen in patients over age 17 and with tumors larger than 5 cm. Eilber et al. have demonstrated an 88% four year disease specific survival for those treated with ifosfamide chemotherapy versus 67% for those who were not [43]. Rosen et al. demonstrated that metastatic synovial sarcoma lesions are particularly sensitive to chemotherapy [44]. In contrast to these studies, there are some that have noted no definitive benefit of chemotherapy in synovial sarcoma [45]. Pediatric rhabdomyosarcoma, when treated with chemotherapy, has a five year overall survival rate of 71% [46]. Subsets of these patients may even be successfully treated without significant local therapy in the form of surgery or radiation.

Broadly accepted indications for adjuvant chemotherapy include synovial sarcoma and pediatric rhabdomyosarcoma, local recurrence and metastatic disease; these indications generally reflect the use of chemotherapy in our practice. Relative indications for chemotherapy include high-grade deep tumors greater than five centimeters and intermediate-grade tumors greater than ten centimeters [5] especially in younger patients. 

### 3.3. Surgery without Adjuvant Therapy

#### 3.3.1. Definition of Margins

The mainstay of treatment for soft tissue sarcoma is surgical resection. Margins status is usually reported according to the system defined by Enneking et al.: intralesional, marginal, wide and radical [11]. An intralesional margin is defined as having the plane of resection into the tumor itself; biopsy is an intralesional procedure. Marginal margins pass through the pseudocapsule and reactive zone. Wide margins preserve a cuff of normal tissue surrounding the entire tumor and radical margins remove the entire compartment containing the tumor. The standard goal is to remove the whole tumor with a wide margin, although pre-operative radiation with tumor “down-staging” effect or postoperative radiation therapy has allowed has allowed for focally marginal resection to facilitate functional limb salvage surgery equivalent local control rates [35]. In a large cohort of 2084 patients with sarcoma, researchers from Memorial Sloan Kettering Cancer Center have demonstrated that a margin positive for tumor approximately doubles the risk of local recurrence [47]. However, in their analysis they did not evaluate adjuvant treatments utilized. 

#### 3.3.2. Indications for Amputation

Despite the advances in limb salvage surgery there are still indications for amputation in the primary management of soft tissue sarcomas. If amputation is selected, it is generally done without pre- or postoperative radiation. The main indications for limb-ablation are related to tumor size and extent [48]. Tumors that escape their compartments and invade neurovascular bundles are difficult to resect with adequate margins. If the entire neurovascular bundle of an extremity is invaded or surrounded by tumor and must be resected, amputation may be the best option. If a single vessel must be resected with a margin it can often be reconstructed to potentiate limb salvage [49]. If a single nerve must be resected to maintain an adequate margin, it very much depends on the function of the nerve. In the upper extremity it is best to preserve the limb as an even partially functional hand is considered better than a prosthetic limb. In the thigh function is still quite good with sacrifice of either the sciatic [50] or femoral nerve [51]. Below the knee, if the peroneal nerve must be resected limb preservation is still a very good option, as the use of an ankle foot orthosis (AFO) is generally quite well tolerated and very cosmetic. If the tibial nerve must be resected, limb-sparing resection is still a good option. Depending on the actual amount of post-operative deficits a floor-reaction AFO can be considered to substitute for loss of plantar flexion strength. When resections of the sciatic or tibial nerve are performed patients should be counseled to carefully monitor their feet for cuts and sores as they are insensate. Consideration should be given to amputation in the setting of patients with comorbidities of diabetes mellitus or immune compromise. 

The anatomic extent of tumors must be considered as well. Patients with tumors in extra compartmental sites such as the axilla, antecubital and popliteal fossae are at increased risk of amputation because of the lack of anatomic barriers to tumor involvement of critical structures.

In general, the patient should be very involved in the decision making process. A lengthy discussion is often needed to educate the patient regarding the relative functional states and risks of recurrence when considering amputation versus limb salvage and most are more accepting of an outcome in which they played a decision making role. 

#### 3.3.3. Outcomes of Limb-Sparing Surgery Alone

Analysis of the available literature would suggest that certain carefully selected patients may do well with surgical resection alone for management of their soft-tissue sarcoma. An analysis of the experience at the Mayo Clinic demonstrated that in 34 patients treated with surgical resection alone demonstrated an overall local control rate of 80% and overall survival rate of 82% [52]. However, when they analyzed the patients by tumor grade they found that all local and distant recurrences were in patients with high-grade tumors. In that subgroup the local control rate was 60% with a 5-year survival of 69%. These investigators concluded that surgical resection alone for patients with low-grade soft-tissue sarcoma was acceptable provided that in the event of local failure limb-sparing surgery was still possible.

A 1997 study from the University of Chicago Medical Center evaluated the outcome of 62 patients with subcutaneous sarcomas treated with surgical resection with or without adjuvant post-operative radiation [53]. This was a group composed of mostly (95%) patients who had previous unplanned excisions prior to referral. These investigators found that when examining their entire cohort there was an 85% disease-free survival at 5 years. There were three local recurrences, all in patients that had received radiation and a marginal excision, and eight deaths with seven of them occurring in patients receiving radiation. The authors acknowledged that their radiation cohort was biased towards patients with high-grade lesions. However, from this data were able to conclude that surgical resection alone is safe for low-grade subcutaneous sarcomas, even in the setting of a prior unplanned excision.

Researchers from the Dana-Farber Cancer Institute/Brigham and Women's Hospital published results of 74 patients with soft-tissue sarcoma of the extremities or trunk managed with surgery and no radiation [54]. Their cohort consisted of mostly low/intermediate grade (51 of 74) and mostly small (average size 4 cm) tumors. In their series they noted an overall local control rate of 93% at 10 years. When analyzing risk factors for local recurrence the only statistically significant variable was size of closest margin, with an 87% local control rate for those with margins less than 1 cm and 100% for those with greater.

Based on the available literature our practice is to carefully select patients for treatment with surgical resection alone, reserving it for those with small, low-grade lesions which are amenable to complete wide resection.

### 3.4. Surgery in Combination with Adjuvant Therapy

#### 3.4.1. Limb Salvage Surgery

Surgical resection with preservation of the limb has become the standard of care in treatment of soft tissue sarcoma of the extremities. This is in large part thanks to availability of high quality cross sectional imaging and adjuvant treatments, namely external beam radiation therapy. Current MRI technology allows for full evaluation of the tumor extent, compartment location and proximity to neurovascular structures. This provides the surgeon a detailed knowledge of expected margins for pre-operative planning of surgical approach and use of adjuvant therapies. The addition of preoperative radiation has also allowed for further efforts toward limb salvage procedures. As radiation theoretically sterilizes the reactive zone about the tumor it has been demonstrated that marginal excision, when needed near vital structures, can safely be performed without compromising local control rates [35]. If radiation therapy is performed postoperatively, the tumor bed should be parked with metal clips to allow a more precise radiation therapy.

Surgical technique should start with careful pre-operative planning with an MRI to create a map of the desired plane of resection. The skin incision should then be longitudinal following the course of major neurovascular structures to allow for proximal and distal extension if necessary. A wide ellipse of normal tissue around the biopsy should be left in continuity with the tumor. This removes any malignant cells that may have persisted in the biopsy tract. Care should be taken to raise full thickness skin flaps to include the underlying fascia to preserve blood supply if possible. If not, the flaps should be made as thick as oncologic principles allow which minimizes the risk of marginal wound necrosis. Similarly, the surgeon should avoid using forceps on the skin edge, especially following either chemotherapy or radiation therapy. The dissection should then proceed circumferentially around the tumor, leaving a cuff of normal tissue, until the depth of the planned deep resection margin is encountered. At that point the tumor can be mobilized deeply on one end and elevated under tension until it is freed. A tourniquet, if used, must be deflated prior to closure and hemostasis obtained prior to closure. In all but the smallest tumors we prefer to leave at least one deep suction drain in place. Large hematomas and seromas contribute to post-operative wound complications. The drains should exit in line with the skin incision, distally if possible in the event that later amputation is required. They are generally left in place until output is less than 30 milliliters per eight hour shift. Skin is closed in layers with suture appropriate to the particular wound. Consideration should be given to given the high risk nature of these wounds and the potential need to leave sutures in for a prolonged period of time. Again, care should be taken not to compress the wound edges with forceps. 

#### 3.4.2. Outcomes of Limb-Sparing Surgery Combined with Radiation

As discussed in the radiation section of this review, the local control rate of soft-tissue sarcoma combined with radiation is 90% or greater. A series from the University of Florida has demonstrated that local control rates with pre-operative radiation following surgery are equivalent at 95%, even when a marginal margin is required to facilitate functional limb-salvage [35].

Although it was not a primary endpoint of their investigation, O'Sullivan et al. demonstrated in their randomized controlled trial that there was no significant difference in local control or distant metastasis when patients were treated with pre-operative versus post-operative radiotherapy [34]. This group did, however, demonstrate an increased likelihood of late radiation effects such as skin fibrosis, edema, joint stiffness and fracture in the arm treated with post-operative radiation.

Compiling this evidence, the preference at our institution is to treat soft-tissue sarcoma with pre-operative radiation followed by surgical excision. 

#### 3.4.3. Management of Unplanned Excision

The inadvertent removal of a tumor under the presumption that it is benign is termed an unplanned excision. It is estimated that up to 90% of subcutaneous sarcomas are initially treated with unplanned excision [53]. Approximately 49–59% of sarcomas treated with unplanned excision have residual tumor [53, 55, 56]. These patients should undergo tumor bed re-excision and consideration of post-operative adjuvant radiation treatment depending on tumor grade and histologic margin status at the re-excision. Despite no apparent impact on overall patient survival [53, 57], the presence of residual microscopic disease is a risk factor for local recurrence [57], and risk of recurrence is higher than in patients managed by primary wide excision. Many of these patients require soft-tissue coverage by way of a skin graft or flap due to the extensive tumor bed excision [58, 59]; whereas primary closure may have been possible if proper oncologic technique was utilized initially. 

### 3.5. Surveillance

Following definitive treatment of a soft-tissue sarcoma the patient must be followed closely for potential development of local recurrence or metastatic disease. 

With regard to local recurrence we prefer to monitor the site by regular patient self-exams and physician exams of the surgical site at routine intervals. If there is a concern based upon the physical examination or if the patient is felt to be at particularly high risk of local recurrence then an MRI with and without gadolinium enhancement is warranted to evaluate for a potential local recurrence. 

As most metastases are likely to occur within the lung, CT scanning of the chest at routine intervals for surveillance is indicated. For high grade sarcomas, we recommend a CT scan of the chest every three months for the first two years postoperatively, every four months for the third year, and every six months for years four and five. A chest radiograph can then be performed yearly should the patient and physician so desire. For low grade sarcomas it is reasonable to use CT scans selectively for high-risk patients, and chest radiography for those at otherwise low risk of metastatic spread [60].

### 3.6. Local Recurrence

Local recurrence rates after radiation and resection of soft tissue sarcoma have been reported as high as 20% [61, 62]; however, most modern series report the number at 10% or less [34, 35, 63, 64]. Local recurrence can be an extremely difficult problem to manage effectively. There have been multiple reports on treatment strategies which vary from radical resection or amputation to a more conservative limb-sparing re-resection with adjuvant radiation and chemotherapy [63, 65]. Regardless of the approach used, patients who develop a local recurrence of soft tissue sarcoma are believed to have a poor prognosis with regards to local control, metastases and overall survival [64, 65].

### 3.7. Metastases

The overall 5-year survival of patients with metastatic soft-tissue sarcoma is poor. For those presenting with metastatic disease to the lymph nodes, 5-year overall survival is estimated at 23–59% [27, 66–68]. On an individual basis, the prognosis for metastatic disease to the lymph nodes depends on the timing of presentation of the metastatic lesion, with an improved prognosis given to those with metachronous rather than synchronous metastases [68, 69]. Pulmonary metastatic disease in soft-tissue sarcoma carries a 5 year survival rate of approximately 10% or less for patients who are not treated with metastectomy. The 5 year survival elevates to approximately 15–52% for those developing pulmonary metastases after a disease free interval and a metastatic lesion amenable to complete resection [70–72]. Indicators of a good prognosis are single pulmonary metastatic lesions, disease free interval of greater than twelve months, and negative resection margins at the time of the metastectomy [70]. It is for this reason that when metastasis occur as a solitary or few discrete lesions after a prolonged disease free interval, aggressive management by resection or other ablative procedure such as stereotactic radiosurgery is recommended. 

## 4. Summary

Soft tissue sarcoma represents a heterogeneous group of malignancies. Appropriate treatment begins with appropriate staging studies followed by a carefully planned and well-executed biopsy. The biopsy and subsequent treatment should ideally be carried out at a sarcoma center. Treatment plans should be made in a multidisciplinary setting involving input from the surgeon, medical oncologist, radiation oncologist, radiologist and pathologist. Limb salvage surgery is the standard of care; however, there are circumstances in which amputation is necessary or preferred. Radiation therapy in combination with surgical resection is highly effective at achieving local control. The use of chemotherapy is evolving but currently is not well-defined. Patients should be monitored closely after resection of their disease for local recurrence and metastatic spread.

## Figures and Tables

**Table 1a:** (a) AJCC soft tissue sarcoma [10]

Tumor size				
T1		5 cm or less		
T2		>5 cm		

Location				

a		Superficial		
b		Deep		

Lymph nodes				

N0: no nodal metastases				
N1: nodal metastasis present				

Distant metastases				

M0: no distant metastases				
M1: distant metastases present				

Histologic grade				

G1		Low		
G2		Intermediate		
G3		High		

Group/stage	T	N	M	Histologic grade

IA	T1a	N0	M0	G1
	T1b	N0	M0	G1
IB	T2a	N0	M0	G1
	T2b	N0	M0	G1
IIA	T1a	N0	M0	G2, G3
	T1b	N0	M0	G2, G3
IIB	T2a	N0	M0	G2
	T2b	N0	M0	G2
III	T2a, T2b	N0	M0	G3
	Any T	N1	M0	Any G
IV	Any T	Any N	M1	Any G

**Table 1b:** (b) Enneking/MSTS [11] staging criteria

Tumor grade	
Low grade	1
High grade	2

Location	

Intracompartmental	a
Extracompartmental	b

Stage	Description

IA	Low grade, intracompartmental
IB	Low grade, extracompartmental
IIA	High grade, intracompartmental
IIB	High grade, extracompartmental
III	Metastatic (any grade and location)
